# Rifaximin for maintenance therapy in antibiotic-dependent pouchitis

**DOI:** 10.1186/1471-230X-8-26

**Published:** 2008-06-23

**Authors:** Bo Shen, Feza H Remzi, A Rocio Lopez, Elaine Queener

**Affiliations:** 1 The Pouchitis Clinic, Digestive Disease Institute, The Cleveland Clinic, Cleveland, OH 44195, USA

## Abstract

**Background:**

Pouchitis is the most common long-term complication of in patients with restorative proctocolectomy and ileal pouch-anal anastomosis. Patients often develop antibiotic-dependent form of pouchitis requiring long-term antibiotic therapy for remission maintenance. Rifaximin, an oral, non-systemic, broad-spectrum antibiotic with a favorable safety profile, may be a promising candidate agent for maintenance therapy. This historical cohort open-label study investigated the efficacy and tolerability of rifaximin in maintaining symptomatic and endoscopic remission in patients with antibiotic-dependent pouchitis.

**Methods:**

Adult patients with antibiotic-dependent pouchitis received a 2-week course of various antibiotics for induction of remission. Patients in remission then began maintenance therapy with rifaximin 200 mg/day (to 1800 mg/day) for up to 24 months. Pouchitis Disease Activity Index symptom scores were assessed every 1–3 months to evaluate efficacy.

**Results:**

Fifty-one patients began maintenance therapy with rifaximin (median dose 200 mg/day); 33 (65%) maintained remission through 3 months (primary endpoint). Of these 33 patients, 26 (79%) successfully continued maintenance for 6 months after beginning maintenance, 19 (58%) successfully continued for 12 months, and two (6%) successfully continued for 24 months. Only one patient reported an adverse event (transient facial rash).

**Conclusion:**

Patients' response to rifaximin as a maintenance therapy appears to be favorable in this open-labeled trial of antibiotic-dependent pouchitis. Randomized, placebo-controlled trials with a longer follow-up are warranted.

## Background

Restorative proctocolectomy with ileal pouch-anal anastomosis (IPAA) is the preferred surgical treatment for patients with medically refractory ulcerative colitis (UC), UC with dysplasia or cancer, and familial adenomatous polyposis (FAP). Pouchitis, an idiopathic inflammation of the ileal pouch frequently characterized by increased number of loose bowel movements, urgency, and abdominal cramping, is the most common long-term complication of IPAA. [1,2] Up to 50% of patients who undergo IPAA for UC experience at least one episode of pouchitis, whereas few patients (approximately 6%) who undergo IPAA for FAP develop pouchitis. [3-5] The etiology of pouchitis is not well understood but likely involves alterations in luminal bacteria (e.g., bacterial overgrowth) and subsequent dysregulation of inflammatory responses in genetically susceptible patients. [1,3,6] The efficacy of antibiotics and probiotics in treating pouchitis provides additional evidence supporting the role of bacterial alterations in the pathophysiology of this condition. [1,7]

Although many patients with pouchitis experience acute episodes with remission and relapse, up to 17% develop chronic disease that requires long-term therapy for treatment or maintenance. [3,5,8-10] Acute or chronic types of pouchitis can usually be treated effectively with antibiotics, making these agents the mainstay of treatment. Given the role of antibiotics in maintenance therapy, chronic disease is often categorized by how patients respond to antibiotic therapy (i.e., antibiotic-responsive, antibiotic-dependent, or antibiotic-refractory). [2,7] Patients with antibiotic-dependent pouchitis experience frequent (≥ four episodes per year) or persistent, rapidly relapsing episodes that respond quickly to antibiotic therapy but recur soon after discontinuing treatment. Remission maintenance for this type of pouchitis requires long-term, continuous, low-dose antibiotic therapy or frequent full-dose antibiotic pulse therapy. [1-3,11-13] Because long-term or frequent antibiotic therapy can be associated with antibiotic resistance and increased adverse effects with prolonged administration, safely maintaining remission in antibiotic-dependent pouchitis can be challenging. [1]

Rifaximin is an oral, broad-spectrum antibiotic with low systemic absorption (<0.4%) that provides high bioavailability in the gastrointestinal tract with minimal risk of systemic adverse effects. During the nearly 20 years rifaximin has been available in Europe, no clinically significant antibiotic resistance has been observed. [14,15] For patients with chronic refractory pouchitis, combination therapy with rifaximin and ciprofloxacin improved pouchitis disease activity index (PDAI). [6,16] Treatment with rifaximin monotherapy also appeared to improve symptoms in patients with active pouchitis. [17,18]

Given these encouraging preliminary data on the efficacy of rifaximin in pouchitis and concerns about antibiotic resistance and adverse effects with systemic antibiotics, rifaximin may provide an effective option for patients with antibiotic-dependent pouchitis. The present open-labeled, historical cohort study was to investigate the efficacy and safety of rifaximin in maintaining remission in patients with antibiotic-dependent pouchitis.

## Methods

### Patients

This was a historical cohort study. Eligible patients (≥ 18 years of age) with antibiotic-dependent pouchitis were seen in our Pouchitis Clinic between July 2004 and June 2006. As a part of standard of practice, clinical, endoscopic, and histologic data for all patients were entered into the Pouchitis Registry, which was approved by the Institutional Review Board at Cleveland Clinic. Informed consent was provided by all patients.

### Inclusion and exclusion criteria

Patients were required to meet all of the following inclusion criteria: diagnosis of antibiotic-dependent pouchitis, defined as ≥ four episodes per year, each of which responded to a 2-week course of ciprofloxacin or metronidazole but recurred soon after treatment ended; frequent episodes of pouchitis requiring long-term (at least 16 weeks), continuous, low-dose antibiotics or frequent pulse therapy with antibiotics for remission maintenance; and currently symptomatic. Patients were excluded from the study if they had antibiotic-refractory pouchitis (i.e., unresponsive to a 2–4 week course of ciprofloxacin or metronidazole); concurrent cuffitis, irritable pouch syndrome, or Crohn's disease of the pouch; or a prior history of adverse reactions to rifaximin.

### Treatment

**Figure 1:**
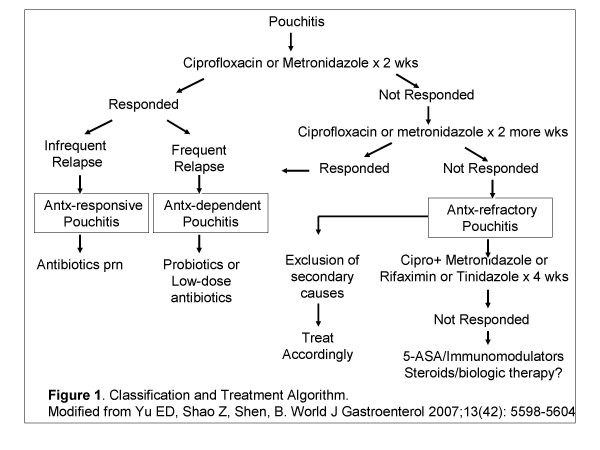
Classification and Treatment Algorithm (Modified from Yu ED, Shao Z, Shen, B. World J Gastroenterol 2007;13(42): 5598–5604).

Management of patients with pouchitis followed an algorithm established for Pouchitis Clinic. The management algorithm was previously published (Figure 1). All patients suspected of having pouchitis underwent clinical evaluation and pouch endoscopy. Symptoms and pouch inflammation were graded using the modified pouchitis disease activity index (mPDAI),[19] which consisted of the symptom (range, 0–6) and endoscopy (range, 0–6) scales from the PDAI. [20] Active pouchitis was defined as mPDAI score >5 points. [19] Patients who had been routinely taking non-steroidal anti-inflammatory drugs (NSAIDs) were asked to discontinue use of these agents for the duration of the study. However, we did not hold the initiation of the antibiotic therapy for induction to allow for wash-out of NSAID use.

To induce remission, patients received single or combination therapy with ciprofloxacin (1000 mg/day), metronidazole (1000 or 1500 mg/day), tinidazole (1000 mg/day), or rifaximin (600, 800, or 1200 mg/day) for 2 weeks. The use of single vs. combination therapy to induce the remission was at the discretion of the treating physician, based on the pattern of patient's prior response to the antibiotic therapy. After the induction period, a repeat pouch endoscopy was performed, and mPDAI scores were determined. Patients who exhibited symptoms or endoscopic signs of pouchitis with mPDAI scores > 5 points were excluded from the maintenance phase of the study; only patients in symptomatic and endoscopic remission began maintenance therapy.

During the maintenance period, all patients received rifaximin at a starting dose of 200 mg/day. As a part of standard care of practice, the patients were followed up in Pouchitis Clinic or contacted via e-mail or telephone every 1–3 months during maintenance (for up to 24 months) to assess symptoms and adverse events and to confirm treatment compliance. Doses of rifaximin were increased (up to 1800 mg/day) for patients who exhibited partial response to maintenance therapy. Treatment was discontinued for patients who were unable to maintain remission with dose escalation or who chose to discontinue therapy. For patients who failed to maintain remission or who chose to discontinue therapy before the end of the 24-month study period, mPDAI symptom scores were documented at the time of discontinuation, and repeat endoscopy was conducted, if possible.

### Primary and secondary endpoints

The primary efficacy endpoint was the number of patients who maintained symptomatic remission for 3 months, as determined by mPDAI symptom scores. Patients who had not maintained remission or exhibited partial response were allowed to continue maintenance therapy beyond the 3-month maintenance assessment. Secondary measures included symptom response to induction therapy, the ability to predict remission maintenance based on clinical factors, and adverse events.

### Statistical analyses

Wilcoxon rank sum, chi-square, or Fisher exact tests were conducted to assess differences between patients who maintained remission for 3 months and those who did not maintain remission for 3 months. Within-group differences between baseline, post-induction, and post-maintenance symptom and endoscopy scores were analyzed using Wilcoxon signed rank tests. Associations between clinical factors and the primary endpoint (remission maintenance at 3 months) were calculated using a multivariate log-binomial model.

## Results

A total of 53 patients with antibiotic-dependent pouchitis were treated with induction therapy with antibiotic monotherapy or combination therapy. Fifty-one patients achieved symptomatic and endoscopic remission during the 2-week induction period and began maintenance therapy with rifaximin. The overall median duration of maintenance therapy was 8 months (range, 0.5–24 months), and the overall median maintenance dose was 200 mg/day (range, 200–1800 mg/day). Following maintenance therapy with rifaximin, endoscopy scores were obtained for 30 (60%) of the 51 patients.

### Remission maintenance

Of the 51 patients who began maintenance therapy, 33 (65%) were still in remission at the 3-month maintenance assessment, and 18 (35%) had relapsed within 3 months. Demographic and clinical characteristics, including age, sex, extent and duration of UC, duration of IPAA, type of pouch, IPAA stage, indication for colectomy, family history of inflammatory bowel disease, smoking, excessive use of alcohol, weekly NSAID use before beginning the study were similar for patients who maintained remission for 3 months and those who relapsed by the 3-month time point (Table 1).

**Table 1:** Demographic and background characteristics

	Remission at 3 months (*n *= 33)	Relapse at 3 months (*n *= 18)	*P*-value
Age, yrs	46.0	47.5	0.6
Male:female, n	18:15	7:11	0.29
Duration of UC, yrs	14.0	12.0	0.79
Type of UC, n (%)			
Pancolitis	31 (94)	16 (89)	0.61
Left-sided colitis	2 (6)	2 (11)	
Stage IPAA, n (%)			0.29
1	0 (0)	1 (6)	
2	25 (76)	15 (83)	
3	4 (12)	2 (11)	
4	4 (12)	0	
Duration of IPAA, yrs	5.0	6.5	0.4
J-type pouch, n (%)	31 (94)	17 (94)	0.99
Family history of IBD, n (%)	7 (21)	5 (28)	0.73
Indication for refractory colectomy, n (%)	23 (70)	17 (94)	0.072
Smoking, n (%)	6 (18)	2 (11)	0.70
Excessive alcohol consumption,* n (%)	3 (9)	0	0.54
Prior weekly NSAID use, n (%)	5 (15)	7 (39)	0.085
Median rifaximin maintenance dose at 3-month assessment, mg/d (range)	200 (200–1800)	200 (200–1200)^†^	0.7
Median duration of maintenance therapy, mo (range)	12 (2–24)	1.3 (0.5–4)	< 0.001
Induction therapy, n (%)			
Monotherapy	27 (82)	8 (44)	NA
Combination therapy	6 (18)	10 (56)	0.006

IBD, inflammatory bowel disease; IPAA, ileal pouch anal anastomosis; NA, not available; NSAID, nonsteroidal anti-inflammatory drug; UC, ulcerative colitis.

*Excessive alcohol use defined as more than one drink per day.

^†^Five of 18 patients who relapsed by 3 months were receiving rifaximin at the 3-month assessment.

As expected, patients who maintained remission for 3 months showed no symptomatic or endoscopic evidence of relapse between the end of induction and the 3-month maintenance assessment (median change 0 points on each PDAI scale; Table 2). For patients who relapsed within 3 months, significant increases in both symptom and endoscopy scores were observed between the end of induction and the 3-month maintenance assessment (median increase of three points on each scale; *P *< 0.0005).

**Table 2:** Symptom and endoscopy scores

Parameter	Remission at 3 months (*n *= 33)	Relapse at 3 months (*n *= 18)	*P-*value
Symptom scores*			
Baseline	4 (3, 4)	4 (3, 4)	0.77
Baseline to end of induction	3 (2, 4)^†^	3 (2, 3)^†^	0.18
End of induction to 3-month maintenance assessment	0	-3 (-3, -2)^†^	< 0.001
Baseline to 3-month maintenance assessment	3 (2, 4)^‡^	0	< 0.001
Endoscopy scores*			
Baseline	3 (2, 3)	3.5 (3, 5)	0.002
Baseline to end of induction	2 (2, 3)^†^	3.0 (2, 4)^†^	0.098
End of induction to 3-month maintenance assessment	0	-3 (-4, -2)^§^	< 0.001
Baseline to 3-month maintenance assessment	2.5 (2, 3)^‡^	1 (0, 1)	< 0.001

*Median (25th, 75th percentiles).

^†^*P *< 0.0001 within-group change.

^‡^Significant within-group change.

^§^*P *< 0.0005 within-group change.

The median total duration of maintenance therapy for patients who were in remission at the 3-month assessment was 12 months, measured from the beginning of maintenance therapy (Table 1). Of the 33 patients who were in remission at the 3-month time point, 26 (79%) continued therapy for 6 months after beginning maintenance, 19 (58%) for 12 months, five (15%) for 18 months, and two (6%) for at least 24 months. Of the 33 patients, 4 had had symptom recurrence sometime between months 3 and 12. Throughout the total maintenance period, the majority of these 33 patients (23 [70%]) received 200 mg/day of rifaximin, whereas 10 patients required dose escalation to 400 mg/day (*n *= 3), 600 mg/day (*n *= 3), 800 mg/day (*n *= 2), 1200 mg/day (*n *= 1), or 1800 mg/day (*n *= 1). In addition, 27 (82%) of the 33 patients who maintained remission for 3 months had received monotherapy during remission induction (Table 1).

As expected, patients who relapsed within 3 months experienced a shorter duration of maintenance than those who maintained remission (median 1.3 months vs. 12 months; *P *< 0.001; Table 1); 13 (72%) discontinued maintenance therapy within 2 months. Patients who relapsed within 3 months had received a median dose of 200 mg/day of rifaximin, a dose similar to that received by patients who maintained remission through 3 months. Eleven (61%) of the patients who relapsed within 3 months had received 200 mg/day of rifaximin, and seven required dose escalation to 400 mg/day (*n *= 3), 600 mg/day (*n *= 3), or 1200 mg/day (*n *= 1) during the total maintenance period. Significantly fewer patients (8 [44%]) who relapsed within 3 months had received monotherapy during the remission induction period compared with patients who maintained remission at 3 months (*P *= 0.006; Table 1).

### Secondary assessments

#### Symptom response to induction therapy

To determine if patients who maintained remission for 3 months responded differently to the initial 2-week induction therapy than those patients who relapsed within 3 months, symptom improvements from baseline to the end of induction were compared. At baseline, symptom scores were the same between responders and non-responders (Table 2). As expected, both patient groups experienced significant symptom improvement from baseline to the end of the induction period (median decrease in mPDAI of three points vs. baseline for each group; *P *< 0.0001), with no significant differences between the two patient groups (*P *= 0.18).

#### Predictors for maintaining remission

Twenty-two variables were analyzed for their ability to predict the efficacy of rifaximin for maintaining remission. Although patients who received antibiotic monotherapy during induction were more likely to maintain remission for 3 months, regression analysis indicated that antibiotic monotherapy during induction was not predictive of maintaining remission for 3 months (Table 3). None of the other variables analyzed, including symptom scores at the end of induction, baseline mPDAI scores, induction doses of antibiotics, and maintenance doses of rifaximin, were predictive of maintaining remission with rifaximin.

**Table 3:** Associations between clinical factors and maintenance efficacy

	Reference	RR (95% CI)	*P*-value
Induction therapy	Single vs. combination therapy	1.67 (0.79–3.49)	0.18
Symptom score after induction	1-unit decrease	1.43 (0.94–2.17)	0.09
Maintenance dose of rifaximin	200-mg/d increase	1.00 (0.87–1.15)	0.97

CI, confidence interval; RR, relative risk.

#### Adverse Effects

Rifaximin was well tolerated when administered for up to 24 months. Only one patient discontinued because of an adverse event (transient facial rash) during maintenance therapy with rifaximin 200 mg/day; this patient discontinued therapy 2 weeks after beginning maintenance.

## Discussion

This open-label study investigated the efficacy of rifaximin (200–1800 mg/day) in maintaining remission in patients with antibiotic-dependent pouchitis. The majority (65%) of patients maintained remission for at least 3 months with rifaximin, indicated by a lack of increase in mPDAI symptom scores. The efficacy of rifaximin in maintaining symptom remission appears encouraging.

Management of antibiotic-dependent pouchitis can be challenging. Because symptoms quickly recur following discontinuation of antibiotic treatments, long-term antibiotic maintenance therapy is often required. Given the potential safety concerns associated with long-term therapy with systemic antibiotics frequently administered for remission maintenance, probiotics have been investigated as antibiotic-sparing agents for maintaining remission in patients with chronic or antibiotic-dependent pouchitis. [11,21,22] Randomized, double-blind trials in Europe showed that VSL#3^®^, a lyophilized bacteria product containing four strains of *Lactobacillus*, three species of *Bifidobacterium*, and *Streptococcus salivarius *subspecies *Thermophillus*, effectively maintained remission for the majority of patients with chronic or recurrent pouchitis. [21,22] However, in an open-label, post-marketing study of VSL#3 in patients with antibiotic-dependent pouchitis which was conducted by our group, only 19% of patients remained on the agents at the end of 8-month trial. [11] These findings suggest that there are barriers in routine use probiotics in this patient population (such as efficacy, concerns of exacerbating symptoms, and cost) and alternative agents are needed for maintenance therapy of antibiotic-dependent pouchitis, particularly in the US patient population.

The present open-labeled study showed that long-term maintenance with rifaximin appeared to be effective in patients with antibiotic-dependent pouchitis, which would provide useful information for our future design of randomized trials. (RVIEWER2 Q2) In contrast, a small randomized trial of oral rifaximin 1200 mg/day vs. placebo (N = 18) showed a marginal therapeutic benefit in treating active pouchitis. [23] The dosage of rifaximin (1200 mg/day) in the study may be too low for treatment of active pouchitis. There are few studies published to date which have examined the efficacy of rifaximin in treating chronic pouchitis. Two studies demonstrated the efficacy of combination therapy with rifaximin and ciprofloxacin on PDAI in patients with chronic antibiotic-refractory pouchitis. [6,16] However, patients in these studies received treatment for only 2 weeks, and efficacy assessments were conducted at the end of treatment to determine the efficacy of rifaximin in inducing remission. Although the study by Abdelrazeq *et al*. [16] included long-term follow-up assessments for pouch failure with pouch diversion or excision, neither of these studies evaluated the efficacy of rifaximin as long-term maintenance therapy. A more recent study (presented in abstract form) in 16 patients with antibiotic- and probiotic-refractory pouchitis demonstrated that 81% of patients achieved symptom remission with rifaximin (600–800 mg/day). [18] The present study extends these findings by demonstrating that rifaximin appeared to be effective for maintaining remission.

Because antibiotic-dependent pouchitis requires long-term, often continuous antibiotic therapy, maintenance treatment and clinical assessments in the present study were extended up to 24 months. Patients were treated with rifaximin as long as they were in remission or until they chose to discontinue therapy. A high percentage (58%) of responsive patients were still on maintenance therapy 12 months after beginning therapy with rifaximin, with two patients continuing maintenance therapy for at least 24 months. These data suggest that rifaximin effectively maintains remission during long-term therapy, extending previous findings. [18]

In addition to being efficacious, long-term treatment with rifaximin was well tolerated. Only one patient reported an adverse event with transient facial rash. The low incidence of adverse events reported in this study is consistent with a 2-week controlled trial for travelers' diarrhea in which rifaximin (up to 600 mg/day) exhibited a safety profile similar to that of placebo, as well as with a 4-month, open-label pouchitis trial with rifaximin (up to 800 mg/day) during which no adverse events were reported. [18,24]

The present study had several limitations, including the open-label historical cohort design, lack of standardized doses of rifaximin throughout treatment, and incomplete endoscopy data for 21 of the 51 patients, a short duration of follow-up (3 month as primary end-point), and loss of follow-up of some patients after 3 months. (RVIEWER2 Q2) Although, in our experience, the dosage required for maintaining remission varied, it is possible that relapse of pouchitis in some patients might result from under-dosing. We chose to start the agent for maintenance therapy with a small dose for its cost and potential risk for bacterial resistance after long-term use. A dose ranging study is warranted for both induction and remission for pouchitis. In addition, the follow-up was set at 3 months after beginning maintenance therapy, although patients could continue maintenance therapy with rifaximin up to 24 months. While construction of Kaplan-Meier curves and estimation of recurrence rates would have been a great addition to this analysis, we unfortunately did not have the information necessary to do time-to-event analysis. In this study, recurrence of symptoms was assessed exactly 3 months after induction therapy for all patients and the exact time of recurrence was not available. Further investigations with prolonged follow-up are needed to more adequately determine the efficacy of rifaximin for maintaining endoscopy remission and for maintenance therapy beyond 3 months.

## Conclusion

Patients' response to rifaximin as a maintenance therapy appears to be favorable in this open-labeled trial of antibiotic-dependent pouchitis. Randomized, placebo-controlled trials with a longer follow-up are warranted.

## Competing interests

Cleveland Clinic maintains policies requiring that certain disclosures of financial interests accompany manuscripts submitted for publication. These financial interests with companies must be disclosed by co-authors from Cleveland Clinic whose research is sponsored by the companies or whose products (or direct and primary competitor's products) are discussed in the manuscript.

This study was supported by the internal fund. However, n accordance with this Cleveland Clinic policy, we are disclosing that we have served within the past year or will serve in the coming year in the following roles in connection with the companies listed below.

**Table 4:** Following roles in connection with the companies.

	Role	Company
Bo Shen, MD	Honoraria	UCB, Centocor, Salix, Abbott
	Research Grant	Ocera
Elaine Queener, LPN	Research Support	Ocera

All authors declared no non-financial competing interest.

## Authors' contributions

BS: Concept, study design and execution, patient recruitment, data entry, and preparation of manuscript. FHR: Concept, patient recruitment, and manuscript preparation. ARL: Data analysis and manuscript preparation. EQ: Patient recruitment, follow-up, and data entry.

## Pre-publication history

The pre-publication history for this paper can be accessed here:



## References

[B1] Pardi DS, Sandborn WJ (2006). Systematic review: the management of pouchitis. Aliment Pharmacol Ther.

[B2] Shen B, Fazio VW, Remzi FH, Lashner BA (2005). Clinical approach to diseases of ileal pouch-anal anastomosis. Am J Gastroenterol.

[B3] Cheifetz A, Itzkowitz S (2004). The diagnosis and treatment of pouchitis in inflammatory bowel disease. J Clin Gastroenterol.

[B4] Lovegrove RE, Tilney HS, Heriot AG (2006). A comparison of adverse events and functional outcomes after restorative proctocolectomy for familial adenomatous polyposis and ulcerative colitis. Dis Colon Rectum.

[B5] Simchuk EJ, Thirlby RC (2000). Risk factors and true incidence of pouchitis in patients after ileal pouch-anal anastomoses. World J Surg.

[B6] Gionchetti P, Rizzello F, Venturi A (1999). Antibiotic combination therapy in patients with chronic, treatment-resistant pouchitis. Aliment Pharmacol Ther.

[B7] Mahadevan U, Sandborn WJ (2003). Diagnosis and management of pouchitis. Gastroenterology.

[B8] Meagher AP, Farouk R, Dozois RR, Kelly KA, Pemberton JH (1998). J ileal pouch-anal anastomosis for chronic ulcerative colitis: complications and long-term outcome in 1310 patients. Br J Surg.

[B9] Lohmuller JL, Pemberton JH, Dozois RR, Ilstrup D, van Heerden J (1990). Pouchitis and extraintestinal manifestations of inflammatory bowel disease after ileal pouch-anal anastomosis. Ann Surg.

[B10] Hurst RD, Molinari M, Chung TP, Rubin M, Michelassi F (1996). Prospective study of the incidence, timing and treatment of pouchitis in 104 consecutive patients after restorative proctocolectomy. Arch Surg.

[B11] Shen B, Brzezinski A, Fazio VW (2005). Maintenance therapy with a probiotic in antibiotic-dependent pouchitis: experience in clinical practice. Aliment Pharmacol Ther.

[B12] Madden MV, McIntyre AS, Nicholls RJ (1994). Double-blind crossover trial of metronidazole versus placebo in chronic unremitting pouchitis. Dig Dis Sci.

[B13] Mimura T, Rizzello F, Helwig U (2002). Four-week open-label trial of metronidazole and ciprofloxacin for the treatment of recurrent or refractory pouchitis. Aliment Pharmacol Ther.

[B14] Koo HL, DuPont HL (2006). Current and future developments in travelers' diarrhea therapy. Expert Rev Anti Infect Ther.

[B15] Scarpignato C, Pelosini I (2006). Experimental and clinical pharmacology of rifaximin, a gastrointestinal selective antibiotic. Digestion.

[B16] Abdelrazeq AS, Kelly SM, Lund JN, Leveson SH (2005). Rifaximin-ciprofloxacin combination therapy is effective in chronic active refractory pouchitis. Colorectal Dis.

[B17] Baidoo L, Kundu R, Su C (2005). Rifaximin is an effective antibiotic for the treatment of pouchitis [abstract]. Gastroenterology.

[B18] Kornbluth A, Hunt M, George J, Legnani P (2006). An open label pilot trial of rifaximin in the treatment of patients with refractory pouchitis [abstract]. Gastroenterology.

[B19] Shen B, Achkar JP, Connor JT (2003). Modified pouchitis disease activity index: a simplified approach to the diagnosis of pouchitis. Dis Colon Rectum.

[B20] Sandborn WJ, Tremaine WJ, Batts KP, Pemberton JH, Phillips SF (1994). Pouchitis after ileal pouch-anal anastomosis: a Pouchitis Disease Activity Index. Mayo Clin Proc.

[B21] Gionchetti P, Rizzello F, Venturi A (2000). Oral bacteriotherapy as maintenance treatment in patients with chronic pouchitis: a double-blind, placebo-controlled trial. Gastroenterology.

[B22] Mimura T, Rizzello F, Helwig U (2004). Once daily high dose probiotic therapy (VSL#3) for maintaining remission in recurrent or refractory pouchitis. Gut.

[B23] Isaacs KL, Sandler RS, Abreu M (2007). Crohn's and Colitis Foundation of America Clinical Alliance. Rifaximin for the treatment of active pouchitis: a randomized, double-blind, placebo-controlled pilot study. Inflamm Bowel Dis.

[B24] DuPont HL, Jiang ZD, Okhuysen PC (2005). A randomized, double-blind, placebo-controlled trial of rifaximin to prevent travelers' diarrhea. Ann Intern Med.

